# The valuation of older adult homecare services under a joint medical-social budgetary perspective

**DOI:** 10.3389/fpubh.2024.1428130

**Published:** 2024-12-24

**Authors:** Eman Leung, Jingjing Guan, Amanda M. Y. Chu, Sam C. C. Ching, Yilin Liu, Frank Youhua Chen

**Affiliations:** ^1^Department of Management Sciences, City University of Hong Kong, Hong Kong, Hong Kong SAR, China; ^2^JC School of Public Health and Primary Care, The Chinese University of Hong Kong, Hong Kong, Hong Kong SAR, China; ^3^Epitelligence, Hong Kong Special Administrative Region of China, Hong Kong, Hong Kong SAR, China; ^4^Department of Social Sciences and Policy Studies, The Education University of Hong Kong, Hong Kong, Hong Kong SAR, China

**Keywords:** homecare, generalized cost-effectiveness analysis, allocative efficiency, ageing in place, integrated care

## Abstract

**Background:**

Homecare, a cornerstone of public health, is essential for health systems to achieve the Sustainable Development Goal (SDG) of universal health coverage while maintaining its own sustainability. Notwithstanding homecare’s system-level significance, there is a lack of economic evaluations of homecare services in terms of their system-wide cost-savings. Specifically, decisions informed by a joint medical-social budgetary perspective can maximize the allocative efficiency of assigning a diverse service mix to address the complex needs of the older adult population. However, little is known regarding which homecare service mix is most system-wide cost-effective when paired with which clinical profiles.

**Methods:**

Valuation of homecare’s complex interventions was performed under a generalized cost-effectiveness analysis (GCEA) framework with proportional hazard-adjusted metrics representing the common numeraire between medical and social care.

**Results:**

Instrumental homecare, on its own or combined with either one or both of the other homecare services, yielded the greatest cost savings compared to other services or the lack thereof. When expressed under a joint medical-social budgetary perspective, instrumental homecare can reduce medical costs of HK$34.53 (US$4.40) and HK$85.03 (US$10.84) for every HK$1 (US$0.13) invested in instrumental and instrumental-restorative homecare, respectively.

**Conclusion:**

Instrumental homecare can increase hospitalization-free days among community-dwelling older adult and yield significant net system-wide cost savings. Thus, the current study demonstrated the feasibility of data-informed decision-making in system-wide resource allocation under a joint medical-social budget perspective.

## 1 Introduction

Public health, as a determinant (1) and measure (2) of Sustainable Development Goals (SDG), is critical to its pursuit (3), especially in terms of achieving universal health coverage (4). On the other hand, older adult homecare, a cornerstone of public health (5–7), is essential for the achievement of population health for all while enabling sustainable development of the health system (8–10). For example, older adult homecare enables older persons to live independently in the community, reducing older persons’ reliance on institutional care, alleviating the burden on the healthcare system, ensuring timely health access by the broader population, and achieving sustainability of the healthcare system (11, 12).

Specifically, as the population ages, the number of older persons who require assistance in the functional dependencies in activities of daily living (ADLs) or instrumental activities of daily living (IADLs) grows, and the need for restorative care in functional re-enablement increases, especially among the growing number of older persons suffering from disabilities and chronic illness (13). Functional dependencies in ADL or IADL are the key drivers of frequent hospitalizations (14, 15). Hence, not only can homecare services enhance older individuals’ quality of life by assisting older adult individuals’ functional dependency or restoring their independence, but effective homecare services can also lengthen the hospitalization-free time the older persons spend in the community (or delay or prevent long-term care utilization) and thus achieve ageing in place (13, 16).

Knowing that homecare is effective is not enough to enable efficient allocation of scarce healthcare resources. We also need to know which homecare service is most cost-effective under what circumstances to inform decisions that can advance public health and achieve long-term viability (17) and sustainable development (18) of our health system. However, studies investigating the cost-effectiveness of homecare services have yielded mixed results. Not only do the reported homecare interventions delivered multipronged service ensembles to client populations diverse in clinical and service utilization histories, but the effectiveness of these services is also heterogeneous, depending on the matches between service types and client characteristics studied (19–22).

Hence, given the heterogeneity, homecare services’ cost-effectiveness may not be able to adequately evaluated by the traditional methods of comparing a single intervention to the current gold standard. In contrast, cost-effectiveness analysis performed under an allocative efficiency perspective is more aligned with the objective of valuating complex intervention where services are multipronged and ensembled, costs are varied, profiles of service recipients are diverse, and individual service outcomes are heterogeneous (23, 24).

In addition, research has also demonstrated the benefit of performing cost-effectiveness analysis under an allocative efficiency perspective. For example, service decision informed by cost-effectiveness analysis performed under an allocative efficiency perspective significantly decreased service costs and improved outcomes (25). The benefit is most pronounced in decisions related to social care where heterogeneity is the greatest (26). Another benefit of performing cost-effectiveness analysis under an allocative efficiency perspective is that it enables a joint medical-social budgetary approach to cost-effectiveness analysis, which is a recommended economic evaluation method for social services, when costs and outcomes are defined in the context of what scarce resources are available to be allocated across different sectors of our society and the transdisciplinary benefits of their allocations (27).

Nevertheless, while cost-effectiveness analyses conducted from a joint medical-social budgetary perspective are lacking among the published economic evaluations of homecare, such an analysis can inform decisions that maximize cross-sector allocative efficiency in tailoring diverse homecare service mix to the complex needs of the older adult population, which can, in turn, mitigate challenges that population ageing posts to the attainment of the sustainability develop the goal of universal health coverage. Hence, with a joint medical-social budgetary perspective, we performed an evaluation of homecare service under a Generalized Cost-Effectiveness Analysis (CGEA) framework to assess the allocative efficiency of individual homecare services by comparing different combinations of homecare services’ medical cost savings, parameterized as common monetary values. Specifically, the homecare services’ medical cost savings were Cox’s model adjusted by homecare recipients’ clinical and functional risk of hospitalization to achieve a more accurate estimate of homecare services’ value for money.

## 2 Method

### 2.1 Study setting

The current study consisted of 633 clients of a homecare service who consented to the service receiving their discharge summaries issued by hospitals in the same catchment area. The studied homecare was a standard service delivered by one of the sixty NGOs commissioned by the social care bureaucracy of Hong Kong (HK)’s publicly funded healthcare system. It is worth noting that HK’s healthcare system also provides medical care under a separate bureaucracy. The mandated objective of the studied homecare services is to facilitate “ageing in place” among clients who are otherwise capable of living independently in the community despite transient unfavorable circumstances. At intake, a licensed social worker assessed the clients’ needs, based on which the clients were assigned one or more personal, instrumental, and restorative services.

Personal homecare offered assistance with bathing and general domestic duties. Instrumental homecare assisted with shopping (e.g., purchase and delivery of daily necessities), food preparation (e.g., provision of meals), transportation (e.g., escort to clinics), managing finances (e.g., application of financial assistance), and housekeeping. Finally, restorative homecare managed and prevented different clinical issues, including accidents and falls, wounds and pressure injury, medications, diet and nutrition, cognitive impairment, convalescent, infection control, chronic pain, depression, agitation/aggressive behaviors, constipation, incontinence, etc.

All participants were assigned at least one homecare service. However, not everyone’s service package had commenced within the study period. As a result, some participants have no recorded service transactions on file.

### 2.2 Data and variables

Data extracted from the hospital discharge summaries included the timestamps, types, and duration of each medical encounter and the client’s corresponding medical diagnoses. On the other hand, data extracted from homecare clients’ records were timestamps of when a homecare service was received, and the type(s) of homecare services received at each transaction. Each client’s data resulting from their medical and social care were linked and sequenced together with respect to the chronology of timestamps. Consequently, a dataset was created to host data generated at both the inter-individual and intra-individual levels.

The outcome variable was the time between consecutive medical encounters (for example, days between the convalescent care from which a client was discharged and the subsequent acute care to which a client was admitted). Post-discharge records were right-censored if no subsequent admission to medical care was found. Explanatory variables were the types of homecare services the client received between consecutive medical encounters (the service’s duration and frequencies were used for calculating the total homecare cost, see below). To adjust confounds from estimating each type of homecare service’s effect on the number of days between consecutive medical encounters, we included the following covariates: The clients’ demographics, medical history of chronic illnesses, licensed social worker-assessed functional impairment, emotional and cognitive issues, and the presence of chronic risk factors such as smoking. Also adjusted at covariates were the types and intensity of the acute and post-acute follow-up services utilized.

### 2.3 Calculation of medical and homecare costs

The cost of each medical encounter of a client was calculated by totaling the published cost of each service utilized during this medical encounter. For example, the published average cost of each A&E attendance was HK$1,230 (US$159; exchange rate of US$1 to HK$7.75), each bed-day at the acute inpatient ward was HK$5,490 (US$708), and each bed-day at a convalescent hospital was HK$2,390 (US$308) in 2015/16 (28). Consequently, the medical cost for a homecare client who spent 3 days at an acute hospital followed by 2 days at the convalescent hospital is HK$22,480 (US$2,899). To align with homecare costs, we adjusted the medical costs with the inflation rate using the Composite Consumer Price Index of the Census and Statistics Department of HK.

The unit costs of each of the three homecare services have not been made public by the social care bureaucracy. Hence, we estimate the unit cost of each service type from two components: One that is unique to each service and one shared by all three service types. Service-specific costs were calculated from each professional category’s published median hourly rate to which service-specific staff belonged and the number of hours spent delivering each service. Shared costs were estimated by deducting service-specific costs from the total homecare budget, derived from the HK government’s annual budget allocated to service operators for each homecare client across sixty NGOs (28).

### 2.4 Analysis

With the number of hospital-free days spent in the community since the last hospital discharge as an outcome, a Cox’s proportional hazards model (29) was built from the utilization metrics of different types of homecare services received between medical encounters as explanatory variables. In addition, our modeling of hospitalization outcomes from homecare utilization was adjusted for the effects of the following covariates: Clients’ medical/functional characteristics and the types of medical care received since the last discharge. In addition, a gamma frailty term (30) was added to the Cox’s model to account for the effects of individual differences in deterioration rate on hospitalization outcome. Consequently, Cox’s frailty model estimated, in terms of risk-adjusted hazard ratios (HR), each homecare service’s marginal “survival benefit,” expressed as the reduction of hospitalization risk by an amount equal to 100*(1-HR)% (31).

Furthermore, log-likelihood ratio (LR) tests were performed to demonstrate that Cox’s frailty model was superior in data fitting to one without the frailty term or a null model with no explanatory variable when modeling the “survival benefit” of older adult homecare services. In addition, to assess the performance of the predictive models and their respective features, concordance statistics (c-statistics) of the Cox’s frailty model and the single-level Cox model were computed, respectively, and compared. A c-statistic between 0.60 and 0.70 indicated fair to modest performance, and a concordance of 0.80 and above indicated excellent performance (32).

Several properties of Cox’s model and the resulting HRs are especially relevant to our valuation of homecare’s complex interventions under a GCEA framework.

For example, in the counterfactual scenario that serves as the reference through which different combination of homecare services’ effects were compared to one another, the subgroup that received no homecare service was assigned an HR of “1″ and thus served as the baseline for risk reduction (i.e., 100*(1-HR)% = 0%) to which those who received different compositions of homecare were compared.

In addition, the reference value of HR = 1 could be assigned to any service combinations of homecare and be chosen as the counterfactual scenario, depending on the which services were the target of comparison. In addition, “*r*” is advanced here to parameterize the risk-reduction effects of the multiple services in homecare’s complex interventions with respect to its corresponding baseline scenario. Specifically, *r* is the product between the risk-adjusted HR of a composition and its baseline HR, which may be something other than one if the counterfactual baseline consists of HR > = 1.

Hence, in addition to the no-service (or selected-service) counterfactual scenario, whose HR was the reference value from which the corresponding service compositions’ *r*s were calculated, each homecare composition also has its counterfactual scenario in estimating its related medical costs. The counterfactual medical cost refers to the medical cost that recipients of specific homecare composition would have incurred if they had not received the assigned homecare composition. Hence, the counterfactual medical cost (M_a_) of each homecare composition was calculated by adjusting off homecare’s reduction of hospitalization risk (*r*) from its associated observed medical cost (M_0_), i.e., M_a_ = M_0_/r. Consequently, medical cost saving is noted (∆M > 0, where ∆M = M_a_-M_0_) when the risk of hospitalization outcomes is reduced among those who received homecare relative to those who did not (*r* < 1). On the other hand, no medical cost-savings (∆M = 0) could be concluded if *r* = 1 and additional medical costs had been incurred (∆M < 0) if *r* > 1.

Finally, to examine net cost-savings from a joint medical-social budget perspective, we developed a ratio representing homecare’s medical cost savings relative to the cost of homecare. Specifically, homecare cost was calculated by first tallying the unit cost of each homecare service offered every time during medical encounters (t), then taking the median of all between-medical-encounter total costs. Since not all counterfactual medical costs were calculated with reference to a scenario that lacks any homecare service, the net cost-saving ratio presented here is calculated by dividing from the medical cost savings (i.e., ∆M) the difference between the counterfactual scenario’s homecare cost and the cost incurred from specific homecare composition of interest (∆S(t)). Consequently, the ratio of net cost saving is E = ∆M/ ∆S(t).

In addition, notwithstanding the clients were also recipients of other homecare services concurrently, restorative homecare was generally assigned to those suffering from higher medical and social needs. Hence, separate cost-effectiveness analyses were performed under the GCEA framework for those who received restorative homecare and those who did not.

## 3 Results

### 3.1 Descriptive statistics

Table 1 shows the characteristics of the study sample (*n* = 633), of which 55.0% were female, and over 70% of records were from participants aged 80 or above. The clients’ average time spent in the community between hospitalizations was 179.10 days. Around 30.8% of the records were right-censored.

**Table 1:** Characteristics of the study sample (*n* = 633).

Variable	Level	Percentage of study sample or mean (standard deviation) [min, median, max]
Restorative Home Care	Absent	55.5%
	Present	44.5%
Personal Home Care	Absent	86.1%
	Present	13.9%
Instrumental Home Care	Absent	24.8%
	Present	75.2%
Type of Last Discharge Hospital	Acute	88.6%
	Non-acute	11.4%
Day Hospital or Outpatient Clinic Follow-up	No	95.6%
	Yes	4.4%
Previous Length of Stay in Acute Hospital, days		6.48 (13.62) [0, 2.97, 251.03]
Previous Length of Stay in Non-Acute Hospital, days		1.31 (4.77) [0, 0, 44.98]
Discharge Specialty of Previous Medical Admission	Medicine and Geriatrics	62.4%
	Accident and Emergency	13.0%
	Orthopedic	11.1%
	Surgical	9.6%
	Others	3.9%
Age Group, years	65–74	11.7%
	75–79	14.7%
	80–84	36.3%
	85–89	21.7%
	90 or Above	15.6%
Chronic Risk Factor Presence	No	49.9%
	Yes	50.1%
Chronic Disease Presence	No	60.7%
	Yes	39.3%
Functional Impairment Presence	No	70.0%
	Yes	30.0%
Emotional or Cognitive Problem Presence	No	92.3%
	Yes	7.7%
Gender	Female	55.0%
	Male	45.0%

43.4% of records documented the utilization of two or more types of homecare services:

1. The utilization of personal homecare is documented in 13.9% of records, with an average of 57.9 (SD = 116.8, median = 9.5) transactions between consecutive medical encounters.
2. The utilization of instrumental homecare is documented in 75.2% of records, with an average of 36.7 (SD = 144.1, median = 8.0) transactions between consecutive medical encounters.
3. The utilization of restorative homecare is documented in 44.5% of records, with an average of 59.3 (SD = 227.5, median = 6.5) transactions between consecutive medical encounters.

### 3.2 Model fit and parameter estimates

Cox’s model with a gamma frailty term significantly outperformed (1) the single-level Cox model without the Gamma frailty term and (2) the null model with no explanatory variables, as demonstrated by our LR tests: log-likelihood = −1622.60 vs. –1856.12 and − 1992.86, respectively, where *p* < 2e−16. Furthermore, when the sensitivity and specificity of the prediction model were examined, the Cox’s frailty model also yielded a better c-statistic (0.81) compared to that of the one-level Cox’s model that lacked the frailty term (0.69). Table 2 illustrates the HRs for explanatory variables (i.e., homecare service utilization) and clinical, functional, and service-utilization covariates. Notably, the frailty term’s unique contribution was significant (Chi-squared = 266.21, *p* < 0.01).

**Table 2:** Parameters estimates of the statistical models.

Variable	Level	Cox’s frailty model					Single-level Cox’s model				
		Coefficient estimate	Hazard ratio	Standard error	*p*-value^#^	Sig.	Coefficient estimate	Hazard ratio	Standard error	*p*-value^#^	Sig.
Restorative homecare *(Ref: Absence)*	Present	−0.19	0.83	0.18	0.294		0.03	1.03	0.12	0.829	
Personal homecare *(Ref: Absence)*	Present	−0.05	0.95	0.25	0.831		−0.25	0.78	0.16	0.108	
Instrumental homecare *(Ref: Absence)*	Present	−1.94	0.14	0.18	<2e-16	***	−1.71	0.18	0.15	<2e-16	***
Type of last discharge hospital *(Ref: Acute)*	Non-acute	0.67	1.95	0.26	0.009	**	0.33	1.40	0.23	0.145	
Day hospital or outpatient clinic follow-up (Ref: No)	Yes	−1.03	0.36	0.30	0.001	**	−0.58	0.56	0.27	0.028	*
Previous length of stay in acute hospital		−0.01	0.99	0.01	0.244		−0.03	0.97	0.01	<0.001	***
Previous length of stay in non-acute hospital		−0.04	0.96	0.02	0.014	*	−0.02	0.98	0.02	0.180	
Discharge specialty of previous medical admission *(Ref: Medicine and Geriatrics)*	Accident and Emergency	−0.33	0.72	0.20	0.093	–	−0.71	0.49	0.17	<0.001	***
	Orthopedic	−0.58	0.56	0.23	0.012	*	−0.44	0.65	0.18	0.015	*
	Surgical	−0.14	0.87	0.22	0.508		−0.28	0.75	0.18	0.109	
	Others	−0.61	0.54	0.34	0.075	–	−0.52	0.59	0.29	0.073	-
Age group *(Ref: 80–84)*	65–74	0.09	1.09	0.33	0.798		0.23	1.26	0.17	0.178	
	75–79	−0.40	0.67	0.23	0.081	–	−0.31	0.74	0.16	0.052	-
	85–89	−0.02	0.98	0.22	0.929		−0.03	0.97	0.15	0.830	
	90 or Above	−0.14	0.87	0.27	0.598		0.13	1.14	0.16	0.419	
Chronic risk factors *(Ref: No)*	Yes	−0.09	0.91	0.21	0.655		0.11	1.12	0.11	0.333	
Chronic diseases *(Ref: No)*	Yes	−0.13	0.88	0.25	0.603		−0.09	0.92	0.13	0.480	
Functional impairment *(Ref: No)*	Yes	−0.29	0.75	0.22	0.188		−0.38	0.68	0.11	0.001	**
Emotional or cognitive problems *(Ref: No)*	Yes	−0.24	0.78	0.35	0.481		−0.28	0.76	0.20	0.155	-
Gender *(Ref: Female)*	Male	0.19	1.21	0.20	0.336		0.40	1.49	0.11	<0.001	***
Individual frailty	Chi-squared	266.21		8.48e −15		***					
Model fit	Likelihood Ratio	740.50			<2e-16	***	273.50			<2e-16	***
	Concordance	0.81					0.69				

^#^*p* < 0.1, *p < 0.05, **p < 0.01, ***p < 2e−16.

Cox’s frailty model examined the marginal contribution of individual homecare services and found that instrumental homecare significantly reduced the immediate risk of hospitalization (risk-adjusted HR = 0.14). Also reflecting a reduction in hospitalization risk, the risk-adjusted HRs of personal and restorative homecare were 0.95 and 0.83, respectively. In contrast, if the frailty term was not controlled for in Cox’s model, the recipients of restorative homecare were those who were at greater risk for hospitalization.

### 3.3 Cost-effectiveness analysis

Table 3 shows the observed and counterfactual medical costs and the medical cost savings resulting from their differences, presented separately for homecare compositions that include restorative homecare and compositions that do not. All homecare compositions on record showed cost savings from their respective counterfactual medical costs and in larger magnitude than their corresponding counterfactual scenarios. In particular, instrumental homecare, on its own or combined with either one or both of the other homecare services, yielded the greatest cost savings (HK$114092.94 (US$14721.67), HK$169909.30 (US$21923.78), and HK$218139.24 (US$28147), respectively, compared to the maximum cost savings of HK$4986.20 (US$643.38) when no instrumental homecare was received).

**Table 3:** Estimated medical cost savings per medical encounter.

(Homecare service compositions)			Sample-based estimates	Model-based estimates		
Restorative	Personal	Instrumental	Median Observed Medical Cost^†^ ( $M_{o}$ ), HK$	*r* (the product of risk-adjusted HRs of all individual services in the composition and baseline)	Counterfactual medical cost ( $M_{a}$ ), HK$	Medical Cost Saving ( $\Delta M=M_{a}-M_{o}$ ), HK$
No	No	No	19768.38	1.00	19768.38	0.00
	No	Yes	18573.27	0.14	132666.21	114092.94
	Yes	No	28392.70	0.95	29887.05	1494.35
Yes	No	No	14871.51	0.83	17917.48	3045.97
	No	Yes	23169.45	0.12	193078.75	169909.30
	Yes	No	18757.61	0.79	23743.81	4986.20
	Yes	Yes	26961.03	0.11	245100.27	218139.24

^† For next medical encounter if present.^

Finally, ratios representing net cost savings under a joint medical-social budget perspective were calculated by dividing medical cost savings by the homecare costs shown in Table 4, with reference to the clients of each homecare composition’s median number of days spent in the community between adjacent hospitalizations. Our analysis reveals that the highest net cost-saving ratios are associated with instrumental homecare, whether received on its own (*E* = 34.53) or concurrently with restorative homecare (*E* = 85.03; whose counterfactual scenario’s net cost-saving ratio is 34.27). However, while the homecare composition involving all three types of services yielded the greatest medical cost-saving (HK$218,139.24; ~US$27,789.47), it is also the most costly (HK$19,218.87; ~US$2,449.14), resulting only in a net cost-saving ratio of 11.35.

**Table 4:** Estimated time spent living in the community and homecare cost per survival.

Homecare services			Quality lifetime estimates	Median homecare cost $\Delta S\left(t\right)$ per survival, HK$ (t*intensity per day*cost per service transaction)			
Restorative	Personal	Instrumental	Average survival time^††^, In days (*t*)	Restorative (a)	Personal (b)	Instrumental (c)	(a) + (b) + (c)
No	No	No	17.29	0.00	0.00	0.00	0.00
	No	Yes	148.98	0.00	0.00	3303.99	3303.99
	Yes	No	28.98	0.00	650.95	0.00	650.95
Yes	No	No	11.95	88.87	0.00	0.00	88.87
	No	Yes	177.39	341.95	0.00	1656.38	1998.32
	Yes	No	12.94	182.47	318.71	0.00	501.17
	Yes	Yes	201.75	2483.47	3713.36	13022.04	19218.87

^†† Average time spent living in the community before next clinical pathway triggered by admission to a hospital.^

## 4 Discussion

We conducted economic evaluation of homecare services using a joint medical-social budgetary perspective and achieved allocative efficiency with our approach, whereby specific parings between homecare service mix and clinical profiles were identified as having greater medical cost savings than others. Specifically, our analysis revealed that instrumental homecare has not only significantly increased the “survival benefit” in reducing the instantaneous hospitalization risk, but it has done so cost-effectively, either on its own or in combination with restorative homecare. When expressed from a joint medical-social budget perspective, the net cost-savings associated with instrumental homecare amount to medical cost-savings of HK$34.53 (US$4.46) and HK$85.03 (US$10.97) for every HK$1 (US$0.13) invested in instrumental homecare alone and an instrumental-restorative homecare composition, respectively. While the homecare composition involving all three types of services amounted to the greatest cost savings, every dollar invested in this composition can only save HK$11.35 (US$1.46) in medical cost-savings and was less cost-effective than the instrumental-restorative homecare composition.

Due to its eligibility criteria, restorative homecare serves older adult clients who suffer from higher medical and social needs. Hence, we performed GCEA on the recipients of restorative homecare separately, even though the marginal contribution of restorative homecare was statistically insignificant after adjusting from the model the contribution of medical and social statuses and the frailty term. Consequently, instrumental homecare is cost-effective regardless of whether restorative homecare is concurrently received, which saves HK$50.76 (US$6.55) more than its counterfactual scenario for every additional dollar invested in instrumental homecare. On the other hand, since the counterfactual scenario of clients who received no restorative homecare is HK$0, instrumental homecare saved HK$34.53 (US$4.46) more than the counterfactual scenario for every dollar invested in instrumental homecare.

In addition, the net cost-saving ratio derived from the joint medical-social budget perspective here also allowed us to quantify the amount of additional investment in homecare required to reduce the burden on medical services to an optimal level. For example, while the average daily occupancy (i.e., its demand) of acute care hospitals in HK was 110% (33), patients’ adverse outcomes and staff turnovers are exacerbated when the occupancy rate exceeds 85% (34) (i.e., its optimal capacity). Despite the 25% shortfall between demand and optimal capacity, the medical system of HK has already spent HK$36,202,849,200 (US$4,671,335,380.65) on acute and post-acute care for older adult persons aged 65+ at the current capacity level. Meanwhile, only HK$1,659,961,860 (US$214,188,627.10) was spent on homecare; even if we were to assume all recipients were aged 65+ (which the majority of them were), it represented only 4.59% of the medical expenditure on the same population. Given the above net cost-saving ratio, whereby each dollar invested in homecare can translate into a medical cost savings of HK$34.53 (US$4.46) to HK$85.03 (US$10.97), the shortfall of 25% medical cost could potentially be made up for with a 9 to 21% increase of the homecare budget, which is currently less than 5% of the medical expenditure. Hence, the analyses performed here under the GCEA can enable service planning under a joint medical-social budget perspective.

Furthermore, the current study also contributed to generating economic evidence for social care, which is generally scarce and, even when available, its interpretation nuanced and context-dependent (35–37). Even more scarce, however, is economic evidence of social care generated from a joint medical-social budget perspective. This whole-system approach to the valuation of social care not only mitigates issues like the nuanced and context-dependent interpretation that the traditional economic evidence for social care requires, but also enhances the translation of this evidence into more informed decisions. To our knowledge, there is another working paper by Xie, Leung, Chen, and Or examining the economic evidence of outbound older adult care in the community using a joint medical-social budgetary perspective. Similarly to our study, Xie et al. (61) also parameterize the number of days free of re-hospitalization as outcome using cox models. However, instead of valuating homecare services that social services delivers to the residences of community-dwelling older adult as in our study, Xie et al. assessed the cost-effectiveness of outbound medical follow-up care delivered to older adults institutionalized at residential care homes by hospitals. And because of the nature of medical follow-up service Xie et al. studied, GCEA was not required in their case. They found that by spending 2,868 HKD per year per person in providing outbound clinical assessment and medical care at residential care homes for older adult, the medical system can save 21,562.8 HKD per year per person in readmission cost.

All-in-all, the lack of economic evidence for social care is in stark contrast with the well-established principles for economic evaluation in medical care (38). Both the UK and US’s publicly funded (39, 40) and market-based systems (41, 42) developed guidelines with medical economic evaluations at its core to achieve cost-effective health and medical practices and policies. In fact, the same principles of economic evaluation have also been incorporated into the development of guidelines for social care (43). However, due to the lack of sufficient economic evidence, social care guidelines are published separately from their economic evidence statements even when economic evidence is available, and these statements provided detail explanations for the caveats and assumptions of the analysis that might influence its findings (44).

Economic evidence is the basis of clinical guidelines and the recommendations that guidelines provide to inform transparent, evidence-based, and value-driven decision making (45). However, recommendations for social care are often made in the absence of economic evidence where expert consensus often serves as the basis for recommendations. In addition, social care guidelines’ recommendations differ from that of clinical guidelines’ in their provision of detailed explanations regarding the available evidence’s limited applicability beyond the specific context within which the service of interest is delivered and the targeted population to which the service is delivered (44). For example, the recommendation for ‘Intermediate care including reablement’ (46) stated that only in certain localities when appropriate healthcare infrastructure is available can home-based intermediate care be a more cost-effective alternative to bed-based intermediate care. In addition, the recommendation on intermediate care was based solely on “economic consideration” rather than formal economic evidence – although the recommendation was consistent with the findings of an earlier systematic review on the cost-effectiveness of home and bed-based respite care (47). Consequently, the adoption of social care guidelines suffers with the lack of evidence needed to enable transparent, evidence-based and value-driven decision-making (48).

There are five crucial elements of economic evaluation. While five elements are fundamental to guideline development, they are challenging to be adopted in social care valuations (37, 44) and thus resulting in the poor adherence of social care guidelines (49). Hence, the current study was designed to bridge the gaps of the missing essential elements in the literature on social care’s economic evaluation. The *first* element in performing economic evaluation for guideline development is to determine the question to be addressed and the corresponding methods of valuation to address the question. A clinical guideline is generally developed with a scope pre-specified by experts, whose intention is to provide guidance to a specific form of clinical service; and with a methodology whose valuation of the service of interest is based on comparing its recipients’ perceived utility with that of the current best practice. However, it has been shown that the traditional utility-based method is incomparable with the valuation of social care’s complex interventions (50), which requires addressing the “broader question” of how their limited budget should be allocated between alternative social care services or between social care services and other sectors’ (37). This is especially true for older adult recipients (51). Nevertheless, cost-utility analysis is still the valuation methodology recommended for social care guideline development (49) as clients’ utility can offer a “common measure” (37) [or a “common numeraire” (52)] for cross-service comparisons.

However, having a “common numeraire” conducive to cross-service comparisons requires social care valuation to overcome challenges associated with the *second* and *third* elements that are fundamental to economic evaluation for clinical guideline development, i.e., defining outcomes and costs, respectively. For example, the costs and outcomes for evaluating medical care are parameterized differently than that of medical care. In terms of cost, social care in all major healthcare systems is publicly funded even when their medical care may be private, thus affecting individual recipients’ perception of the cost they are willing to pay. On the other hand, social care outcomes are usually poorly defined and often unmeasurable unless their long-term and/or cross-sector impacts are considered, making them less immediately evidenced to individual recipients than medical care (37). Consequently, individual recipients are more readily to perceive medical care as having greater utility than social care. However, from the perspective of decision-makers who are required to address the broader question of allocating resources between medical and social care, social care incurs a much lower unit cost, and alleviates the long-term demand for medical care (53). Hence, it was suggested that social care should be evaluated as a society-wide investment, whereby the return on investment is also observable in other sectors, such as the medical sector (37), and its costs and outcome should be parameterized as a common monetary measure under a joint medical-social budget perspective (41, 52).

The *fourth* key element of economic evaluations is the selection of comparators. Comparators in the traditional economic evaluation are the current best practice that seeks to bring about the same intended change within the same context. However, to address the “broader question,” comparators should enable the decision to re-allocate resources among different services designed for, and delivered at, different contexts without having to assume that the comparator is necessarily a sufficiently resourced best option (54). Hence, the generalized cost-effectiveness analysis (CGEA) framework has been put forth to enable decision makers to compare across health services for allocative efficiency, especially apt for services with a social objective (55). Specifically, CGEA’s comparator is a hypothetical “null” scenario, allowing a selected components of a complex intervention to be evaluated independently from the effects of other components or other interventions received concurrently (55). However, while GCEA’s comparator enables the comparison between different combinations of services and their respective reference scenarios, the service recipients’ clinical and functional heterogeneity is not addressed. Given that the recipients’ clinical and functional heterogeneity can affect the nature of services being assigned and the outcome of the assigned services, the accuracy of social care valuation could potentially be affected.

The final element of economic evaluations is one’s ability to combine data on cost and outcome together to tailor recommendations for decision-makers needs. For social care valuation, decision-makers need to look beyond the immediate context within which a service is delivered, often beyond the social sector to source data on costs and outcomes to estimate the value for money of social care. For example, social care minimizes costly hospitalization and enables “ageing in place” and this mitigates the effects of population ageing on our system (56). On the other hand, however, social and medical care also compete for the same pool of scarce and dwindling resources, such as human resources and personal protective equipment (57). Hence, the making of optimal decisions of allocating resources between medical and social care requires that data on social care costs and outcomes, while potentially sourced from different sectors, be linked together and then parameterized into a common measure. Although medical-social data linkage is the basis for addressing the broader cross-sector question under the joint medical-social budget perspective advocated for all structurally different healthcare systems (including those with mixed provision (41, 52), and medical/social care silos), data linkage between social and medical services is rare even for centralized healthcare systems (58). To this end, the current study has contributed to the broader question of cross-sector resource allocation by tackling the five elements of economic evaluation that challenge social care valuation.

The current study has several limitations. For example, the current study may suffer from selection biases. Nevertheless, the following methodological and analytical elements of our study may have mitigated the potential selection bias. First, we included every client whose membership was active during the study period, ensuring that the sampling was comprehensive rather than selective. Second, the eligibility of older adults for homecare services, the assignment of service types, and the intensity with which the assigned services were delivered were all determined by the Service Operating Agreement between the social service agencies and the Social Welfare Department of the Hong Kong government. Specifically, licensed social workers performed standardized needs assessments to ensure that scarce public resources are prioritized according to the needs of the potential recipients of government-subvented service. To enable ageing in place of Hong Kong’s older adult population, licensed social workers responsible for the in-take assessment of subvented homecare services (including the one being studied) decide on the eligibility for, and the type and intensity of, service assignment according to the care needs of potential service recipients and the presence of alternative means, formal or informal, that can meet those needs. Finally, the current study also statistically controlled for potential selection bias in terms of: (1) proxy for the presence of alternate means of meeting care needs and (2) frailty term to adjust for the residual heterogeneity that the model had not been accounted for.

Another limitation is that, while the current economic evaluation is based on the frailty- and risk-adjusted coefficients of the three types of homecare services studied, only the risk-adjusted coefficients of instrumental homecare were statistically significant (both with and without the frailty term included into the model).

The question of whether including statistically insignificant coefficients in economic evaluation would weaken its significance is one that captivates the attention of economic think tank (59) and academic researcher (60) alike. Coefficients of linear models have often served as the metrics for economic analysis, under the assumption that the economic significance of the evaluation that these coefficients have parameterized is predicated on these coefficients being statistically significant. However, it has been argued (59, 60) that all coefficients generated for the intended economic evaluation should be considered together if the true significance of economic impact were to be revealed—regardless of the statistical significance of the individual coefficients the economic evaluation was based.

In alignment with the practice advocated by this school of thought, the current study systematically included all resulting model coefficients and compared the effect of their different combinations within a GCEA framework to assess the three services’ unique and combined economic impact. In fact, the need to consider all coefficients is underscored by the fact that the coefficients of all three service variables were statistically significant when modelled individually, irrespective of whether the frailty term was included (i.e., Univariate model without the frailty term—personal: coefficient estimate = −0.36, hazard ratio = 0.70, standard error = 0.13, *p* = 7.7e−03; instrumental: coefficient estimate = −1.75, hazard ratio = 0.17, standard error = 0.29, *p* < 2e−16; restorative: coefficient estimate = −0.54, hazard ratio = 0.59, standard error = 0.10, *p* = 2.8e−08. On the other hand, when the frailty term was included into the model – personal: coefficient estimate = −0.46, hazard ratio = 0.63, standard error = 0.23, *p* = 4.6e–02; instrumental: coefficient estimate = −2.04, hazard ratio = 0.13, standard error = 0.16, *p* = 1.5e−39; restorative: coefficient estimate = −1.09. hazard ratio = 0.34, standard error = 0.15, *p* = 2.9e−13).

Hence, in conclusion, the significant univariate effect of personal or restorative services disappeared when other services were entered into the model, reflecting the lack of independence of their effects—which the current study controls the lack of independence statistically to isolated the unique effect of each service while examining the combined effect of these services within a GCEA framework.

## Data Availability

The datasets generated and analyzed during the current study are not publicly available as they are the property of the partnering NGO, which is bounded by Hong Kong’s Personal Data (Privacy) Ordinance (Cap. 486) (PDPO), including, but not exclusive to, PDPO’s Guidance Note in Cross-border Data Transfer. In addition, the Research Ethics Committees of the PI’s institution do not allow a third-party transfer of social service client information. Nor do the Ethics Committees permit study PI to make public the social service clients’ information.
